# Supplementary material for: Optimization of satellite-based communication links
Source: PLoS One. 2025 Apr 21;20(4):e0315388. doi: 10.1371/journal.pone.0315388 (PMC12011291; doi:10.1371/journal.pone.0315388)
Supplement: Attachment — Submitted filename: 2nd Round Reviewer response.docx [file pone.0315388.s003.docx]

**Editor’s and Reviewer’s Comments**

The Authors would like to take this opportunity to appreciate 2^nd^ round of Editor and Reviewers Constructive feedback and made sure that all the comments made by the worthy persons are addressed to the best of Author’s knowledge. Please find below Comments by Comments Responses. These are also being incorporated in the revised manuscript uploaded by the Authors.

| **Editor/Reviewers** | **Comments** | **Response** |
| --- | --- | --- |
| **Reviewer #1 Comment** | 1. The author has incorporated all the changes as desired. I am satisfied with the current version of the manuscript, and it is now ready for publication. | The Authors really appreciate the time invested by the Reviewer in making this manuscript up to the level of Publication. The valuable feedbacks from the reviewer will go a long way in carrying out future research enhancement on this area. Please accept our appreciations. |
| **Reviewer # 2 Comments** | 1. Despite the authors' revisions, certain sections still require more practical discussion and improved coherence in the discussion of the results. 2. The discussions regarding both technical and practical aspects, as well as comparisons to recent studies on this topic, need a thorough examination. | As per reviewer suggestions multiple sections are appended and more comprehensive analysis of technical and practical aspects are discussed. A substantial write up is added on **page number 20 line numbers 634 to 677.**  Apart from this there are several lines and paragraph rephrased emphasizing the practical and technical aspects of the proposed algorithm in real world scenario |
|  | 3. For effective comparison of the proposed scheme with the other schemes, the variables of the presented results should start from almost the same steady state. However, this is not the case in this paper. | The Author agree with the reviewer comments and would like to apprise that:  “In order to provide a fair and accurate comparison of the proposed scheme with other existing schemes, the variables such as time taken for switching, initial network load, system latency, and hardware configurations are aligned. This ensures that all systems are evaluated under identical or near-identical conditions, thus providing a robust and effective comparison.” |
