# Supplementary material for: Optimization of satellite-based communication links
Source: PLoS One. 2025 Apr 21;20(4):e0315388. doi: 10.1371/journal.pone.0315388 (PMC12011291; doi:10.1371/journal.pone.0315388)
Supplement: Attachment — Submitted filename: Reviewer response.docx [file pone.0315388.s002.docx]

**Editor’s and Reviewer’s Comments**

The Authors would like to take this opportunity to appreciate Editor and Reviewers Constructive feedback and made sure that all the comments made by the worthy persons are addressed to the best of Author’s knowledge. Please find below Comments by Comments Responses. These are also being incorporated in the revised manuscript uploaded by the Authors.

| **Editor/Reviewers** | **Comments** | **Response** |
| --- | --- | --- |
| **Editor’s Comments** | To improve the manuscript, consider providing more detailed explanations of the underlying principles of the proposed algorithm | A complete new section is added in the revised manuscript under the heading of **“Proposed Algorithm” on Page 06, Line # 205-289.**  Moreover a flow chart of the proposed algorithm is also added as **Figure #01 on Page 08, Line#277** of the revised manuscript. |
|  | Explain how the proposed technique integrates with existing satellite communication technologies. | A complete new section is added in the revised manuscript under the heading of **“Integrating proposed technique with Existing Satellite Technologies” on Page 09, Line # 312-406.** |
|  | Additionally, expanding the discussion on the broader implications and potential applications of this optimization technique across different satellite networks would strengthen the manuscript's contribution to the field. | A complete new section is added in the revised manuscript under the heading of **“Implications and Applications of Proposed Algorithm” on Page 17, Line # 533-640.** |
|  | Clarifying the limitations and future research directions would also provide a more comprehensive perspective for readers. | A complete new section is added in the revised manuscript under the heading of **“Limitations of Proposed Technique” on Page 20, Line # 641-731.**  A complete new section is added in the revised manuscript under the heading of **“Future Work” on Page 22, Line # 732-817.** |
| **Reviewer # 1 Comments** | The methodology heavily relies on the assumption that both the primary and secondary hub stations operate on the same iDirect Evolution Software version. This creates a potential limitation as any software updates or version mismatches could render the proposed solution ineffective. | The Authors appreciate the reviewer highlighting the assumption related to both the primary and secondary hub stations operating on the same iDirect Evolution Software version. We acknowledge this as a potential limitation, however, since the algorithm is developed on running hardware actual system this limitation can be justified and further rectified as discussed **on Page#09, Line # 291-311;** Under the headings of “**Software Version Compatibility & Mitigation of Version Mismatch Risks**” of revised manuscript.  Furthermore a subsection of “**Frame ware Development”** under the section of “**Future Work” on Page #22, Line#733-741,** elaborates in detail how to eliminate such limitations in future extension of the proposed work. |
|  | The study lacks a detailed analysis of the performance impacts or potential network congestion that may arise during the automatic switching process. The research also does not adequately consider scenarios where multiple remote terminals simultaneously initiate the switching process, which could lead to network bottlenecks or increased latency. These limitations suggest that while the proposed solution is promising, further testing and optimization are required to ensure its robustness and scalability in diverse SATCOM network environments. | Authors appreciate reviewer’s insights into the potential performance impacts and network congestion during the automatic switching process. Also acknowledge the fact that further testing is essential to analyze and ensure the scalability and robustness of our proposed solution. All these concerns are addressed under a complete new section added in the revised manuscript “**Future Work” on PAGE #22, Line #732-775.** |
| **Reviewer # 2 Comments** | The methodology section provides a general overview of the processes used. However, it would benefit from a more detailed explanation of why certain methods were chosen over others. For instance, the decision to not use iDirect Global NMS could be further elaborated with a comparison of potential alternatives and their limitations. This will help to justify the proposed approach more convincingly. | Details along with explanation of why certain methods were chosen over others already mentioned under the heading “Hub Station Redundancy in an iDirect Network”. Comparison of the available methodologies is included in “**Table 1”, Page#4, and Line #117-118** in the revised manuscript**.**  Moreover since iDirect Satcom equipment is based on proprietary technologies and algorithms unique to the equipment manufacturer i.e. ST Engineering. The only solution provided by the vendor for implementing automatic shifting of remotes between two Hub Station is the implementation of iDirect Global NMS. Since, no alternatives are available; therefore, comparison has only been made with the possible Manual Switching mechanisms / alternatives. |
|  | The manuscript presents a single case study as validation of the proposed model. While the results are promising, the manuscript would be stronger if it included multiple test cases or scenarios to demonstrate the robustness and generalizability of the model across different conditions. This could include varying environmental factors, network configurations, or hardware setups. | Validation of the proposed model has been carried out on a real iDirect Satcom Network. Since, the facility used for the testing is an operational iDirect Network and testing required disruption of services by Switching ‘Off’ Downstream Frequency Carrier of the Primary Hub Station; therefore, multiple test cases or scenarios could not be implemented as same would have resulted in unavailability of the Satcom Network for users.  However, extensive testing and validation of the proposed switching mechanism under various scenarios and load conditions is being suggested under the section of “**Future Work” on Page#22, Line 732-815**. Ensuring that the proposed automatic switching mechanism performs reliably under all circumstances. |
|  | The results section discusses the time savings achieved through the proposed model. However, a more in-depth quantitative analysis, possibly with statistical validation, could provide stronger evidence of the model’s superiority. For example, comparing the performance metrics across several trials and providing a detailed analysis of the variance would add rigor to the findings. | Validation of the proposed model has been carried out on a real iDirect Satcom Network. Since, the facility used for the testing is an operational iDirect Network and testing required disruption of services by Switching ‘Off’ Downstream Frequency Carrier of the Primary Hub Station; therefore, performance across several trials could not be tested as same would have resulted in unavailability of the Satcom Network for users. However, the methodology was tested for more than 20 times to validate the automatic switching during each test.  Extensive testing and validation of the proposed switching mechanism is being suggested under the section of “**Future Work” on Page#22, Line 732-815**.. This will ensure that the proposed automatic switching mechanism performs reliably under all circumstances. |
|  | The manuscript mentions the development of a Unique Option File for registration of Satcom Modems. More detailed information on the implementation of this file, such as the specific parameters configured and how they interact with the network, would be valuable. Additionally, a flowchart or diagram illustrating the process could enhance reader comprehension. | As suggested by the reviewer key features of the developed solution are further enhanced and elaborated under the section “**Optimized Shifting Model” on Page#04, Line # 130-204.**  Furthermore a complete new section is added in the revised manuscript under the heading of **“Proposed Algorithm” on Page 06, Line # 205-289.**  Moreover a flow chart of the proposed algorithm is also added as **Figure #01 on Page 08, Line#277** of the revised manuscript.    These sections further clarify and elaborate the working mechanism of the proposed technique. |
|  | While the manuscript briefly mentions the need for further development, it lacks a detailed discussion of the current limitations of the proposed model. Identifying areas where the model might struggle or scenarios it may not cover would provide a more balanced perspective. Additionally, outlining concrete steps for future research would guide subsequent studies and demonstrate the authors’ awareness of the field’s evolving challenges. | As per reviewer suggestions:  A complete new section is added in the revised manuscript under the heading of **“Limitations of Proposed Technique” on Page 20, Line # 641-731.**  Complete new section added in the revised manuscript “**Future Work” on PAGE #22, Line #732-775.** |
|  | The manuscript is written in a highly technical style, which may limit its accessibility to a broader audience. Simplifying some of the technical jargon and providing brief explanations of complex terms when first introduced could make the manuscript more inclusive to readers from different backgrounds. | The authors as per reviewer suggestions to the best of their knowledge where required explained Jargons and tried to make entire manuscript easily understandable by providing explanations to complex terms where ever possible in a balanced way. |
|  | The manuscript does not address any ethical or practical considerations related to the deployment of the proposed system in real-world environments. Discussing potential risks, such as data security concerns or operational challenges, would provide a more comprehensive view of the implications of this research. | Implementation of the proposed solution, does not tailor / change iDirect's proprietary technology as well as communication algorithm; therefore, it does not result in a data security concern in addition to existing limitations of iDirect equipment. |
